# A new species of the genus *Nicippe* from Japan (Crustacea, Amphipoda, Pardaliscidae)

**DOI:** 10.3897/zookeys.668.12181

**Published:** 2017-04-12

**Authors:** Sachi Matsukami, Takafumi Nakano, Ko Tomikawa

**Affiliations:** 1 Department of Science Education, Graduate School of Education, Hiroshima University, Higashi-Hiroshima 739-8524, Japan

**Keywords:** COI, cryptic species, Gammaridea, *Nicippe tumida*, North Pacific

## Abstract

A new species of the pardaliscid amphipod, *Nicippe recticaudata*, from off Cape Toi, Japan, is named and described. This is the first record of *Nicippe* Bruzelius, 1859 from the western Pacific coast of the Japanese archipelago. Additionally, nucleotide sequences of nuclear 28S ribosomal RNA and histone H3 as well as mitochondrial cytochrome *c* oxidase subunit I (COI) and 16S ribosomal RNA from the holotype and paratypes were determined. The morphological characteristics and the COI distance values enforced the distinctiveness of *N. recticaudata* **sp. n.** among the known *Nicippe* species. *Nicippe recticaudata* **sp. n.** closely resembles *N. tumida* Bruzelius, 1859 in having a two-dentate posterior margin of usoromite 1. However, the former is distinguished from the latter by the posterior margin of merus of pereopod 4 with 5–6 setae, anterior margin of merus of pereopod 5 with 9–10 setae, and telson with straight inner margin, tapering proximally. A key to the species of *Nicippe* is provided.

## Introduction

The amphipod genus *Nicippe* Bruzelius, 1859 has been recorded from 35–1398 m deep, and currently consists of four species: *N. tumida* Bruzelius, 1859, *N. buchi* Andres, 1975, *N. rogeri* Lörz & Schnabel, 2015, and *N. unidentata* KH Barnard, 1932. Its type species *N. tumida* has been diagnosed by the two-dentate posterior margin of urosomite 1. Originally, this species was described based on specimens collected from Drøbak, Norway (Bruzelius 1859). Later, this species has been recorded from North Atlantic, North Sea, North Pacific, East Pacific, Greenland, Mediterranean Sea, and European Fjords (Karaman 1974).

Contrary to the cosmopolitan cryptic species complex *N. tumida*, the other three species had been recorded from the type localities: *N. buchi* was collected from lava tubes off Lanzarote, North Atlantic Ocean (Andres 1975); *N. rogeri* from the central Chatham Rise, off east New Zealand (Lörz and Schnabel 2015); and *N. unidentata* was recorded from the Palmer Archipelago on the Antarctic Peninsula (Barnard 1932; Biswas et al. 2009).

Recently, unidentified specimens belonging to *Nicippe* were obtained from off Cape Toi, Miyazaki Prefecture, Japan, at a depth of 265–367 m. This is the first record of the genus from the western Pacific coast of the Japanese archipelago. Following a detailed examination of the specimens and their genetic data, these amphipods are described as a new species herein.

## Material and methods

### Sample

The present specimens were collected from off the southern tip of Kyushu (St-12) during a research cruise of the T/S *Toyoshio-Maru* (Hiroshima University) to Kyushu and the Nansei Islands, southwestern Japan in 2006 (Cruise # 2006-03). Specimens were collected with a sledge-net (mouth opening 145 cm × 15 cm, mesh opening 328 µm). The gear was towed along the bottom at a speed of 2 knots for 20 minutes. Samples were immediately fixed and preserved in 99% ethanol on-board ship. In the laboratory, specimens of *Nicippe* were sorted from amphipod samples under a stereomicroscope. For DNA extraction, muscle tissue was removed from the dorsal side of the pleon of each of three specimens.

### Morphological observation

All appendages of the examined specimen were dissected in 70% ethanol and mounted in gum-chloral medium on glass slides under a stereomicroscope (Olympus SZ61). The specimens were examined using a light microscope (Olympus BH2) and illustrated with the aid of a camera lucida. The body length from the tip of the rostrum to the base of the telson was measured along the dorsal curvature to the nearest 0.1 mm. The specimens are deposited in the Tsukuba Collection Center of the National Museum of Nature and Science, Tokyo (NSMT) and the Zoological Collection of Kyoto University (KUZ).

### PCR and DNA sequencing

The extraction of genomic DNA from pleon muscle followed Tomikawa et al. (2014). Primer sets for the PCR and cycle sequencing (CS) reactions used for histone H3 (H3) and 16S rRNA in this study were shown in Tomikawa et al. (2016). Those for the other regions were as follows: for 28S rRNA (28S), 28F (Hou et al. 2007) and 28SR (Tomikawa et al. 2012) (PCR and CS) with 28SF (Tomikawa et al. 2012), 28SFNici772 (5'-CCCGGATCGAAATCAGTAG-3'; this study) and 28SRNici679 (5'-CCATAAATTCGACACAGTAG-3'; this study) as internal primers for CS; and for cytochrome *c* oxidase subunit I (COI), LCO1490 and HCO2198 (PCR and CS) (Folmer et al. 1994).

The PCR reaction and DNA sequencing were performed using the modified methods mentioned in Nakano (2012) with the aid of a T100 Thermal Cycler. The PCR reaction mixtures were heated to 95°C for 5 min, then followed 35 cycles as below, and a final extension at 72°C for 6 min: 35 cycles for 28S, at 94°C (10 s), 50°C (20 s), and 72°C (1 min); for H3, at 94°C (10 s), 52°C (20 s), and 72°C (24 s); for COI, at 94°C (10 s), 46°C (20 s), and 72°C (42 s); and for 16S, at 94°C (10 s), 50°C (20 s), and 72°C (30 s). The sequencing mixtures were heated to 96°C for 2 min, followed by 40 cycles at 96°C (10 s), 50°C (5 s), and 60°C (54 s). The obtained sequences were edited using DNA BASER (Heracle Biosoft S.R.L.). The DNA sequences newly obtained in this study were deposited with the International Nucleotide Database Collaboration (INSDC) through the DNA Data Bank of Japan.

### COI genetic diversity calculation

To calculate genetic diversity between the present specimens and the other *Nicippe* sample, one COI sequence (CMBIA134-11.COI-5P) of the amphipod identified as *N. tumida* was obtained from BOLD (Ratnasingham and Hebert 2007). The individual was collected from the eastern coast of the Pacific Ocean (Palos Verdes Peninsula, California, U.S.A.).

The COI sequences were manually aligned, because no indels were observed. Pairwise comparisons of uncorrected *p*-distances for three COI sequences obtained in this study (658 bp) and that obtained from BOLD (651 bp) were calculated using MEGA7.0.16 (Kumar et al. 2016). All missing positions were eliminated for each sequence pair.

## Taxonomy

### Family Pardaliscidae Boeck, 1871

#### Genus *Nicippe* Bruzelius, 1859

New Japanese name: Miko-yokoebi-zoku

##### Nicippe recticaudata sp. n.

Taxon classificationAnimaliaAmphipodaPardaliscidae

http://zoobank.org/811C54A4-0339-4043-809D-2E0715BA7C5A

New Japanese name: Toyotamamiko-yokoebi

**Figure 1.**
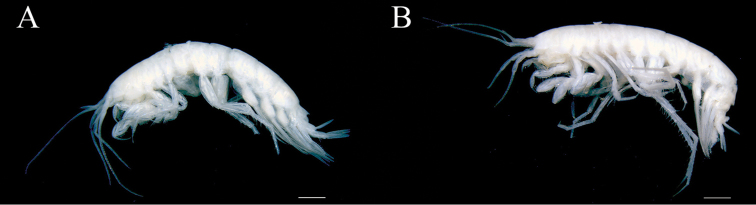
*Nicippe recticaudata* sp. n., habitus, lateral views. **A** holotype, male, 8.4 mm, NSMT-Cr 25456 **B** paratype, female, 8.5 mm, NSMT-Cr 25457. Scale bars: 1.0 mm.

**Figure 2.**
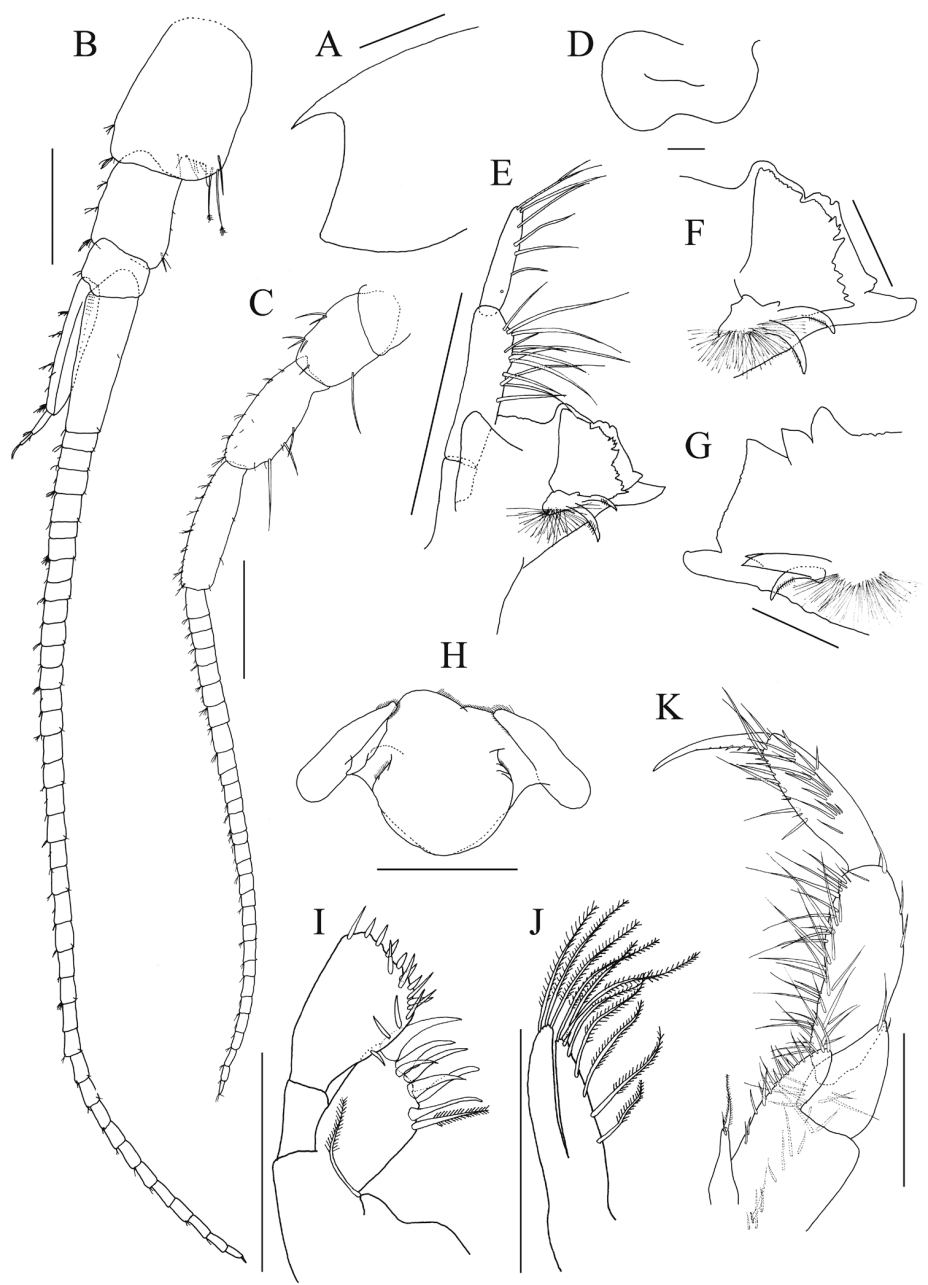
*Nicippe recticaudata* sp. n., holotype, male, 8.4 mm, NSMT-Cr 25456. **A** head, lateral view **B** antenna 1, lateral view, facial setae on accessory flagellum 1 omitted **C** antenna 2, lateral view **D** upper lip, anterior view **E** left mandible, medial view **F** distal part of left mandible, lateral view **G** distal part of right mandible, lateral view **H** lower lip, ventral view **I** maxilla 1, dorsal view **J** Maxilla 2, dorsal view **K** maxilliped, dorsal view. Scale bars: 0.5 mm (**A, E, H–K**), 1.0 mm (**B, C**), 0.1 mm (**D, F, G**).

**Figure 3.**
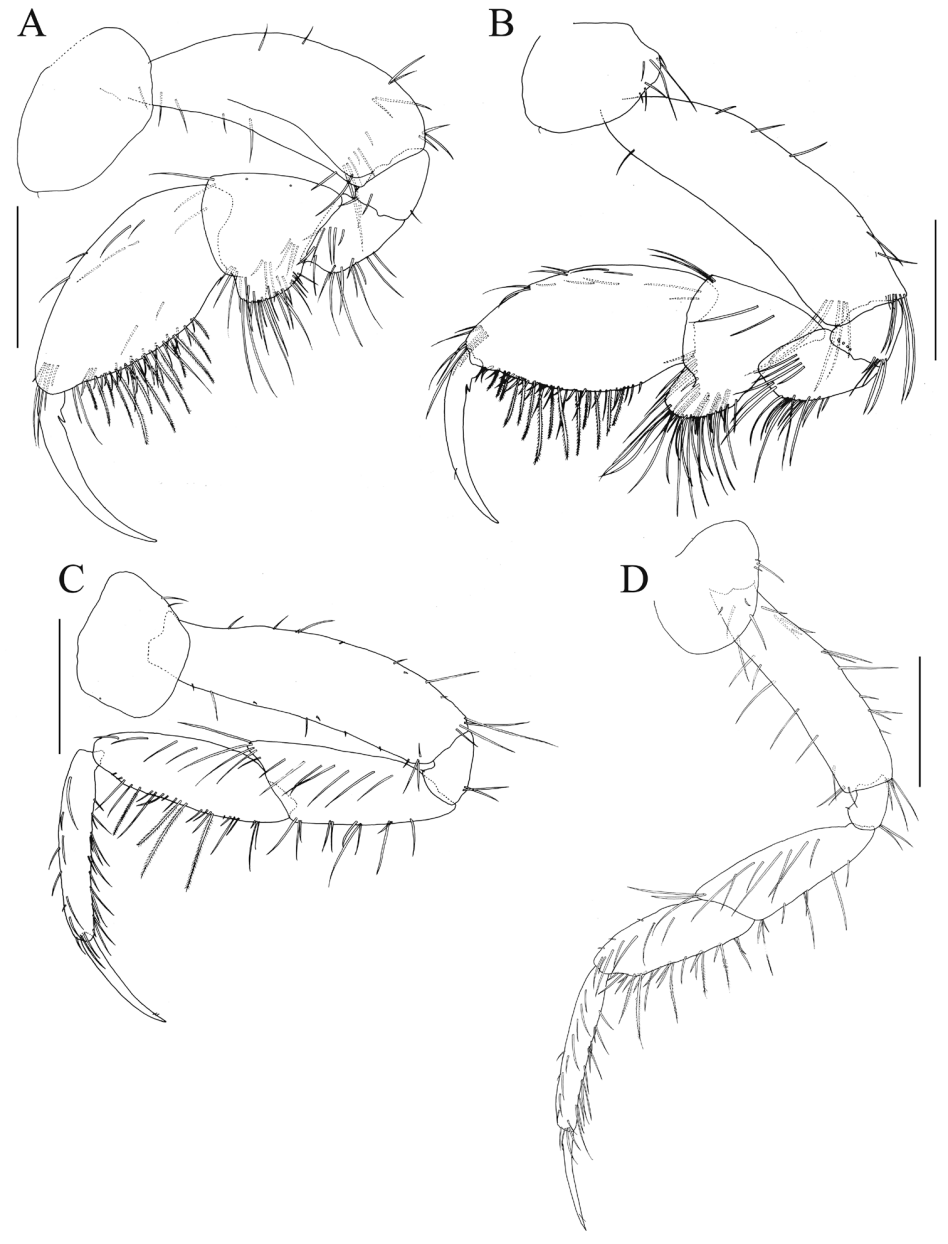
*Nicippe recticaudata* sp. n., holotype, male, 8.4 mm, NSMT-Cr 25456. **A** gnathopod 1, lateral view **B** gnathopod 2, lateral view **C** pereopod 3, lateral view **D** pereopod 4, lateral view. Scale bars: 0.5 mm.

**Figure 4.**
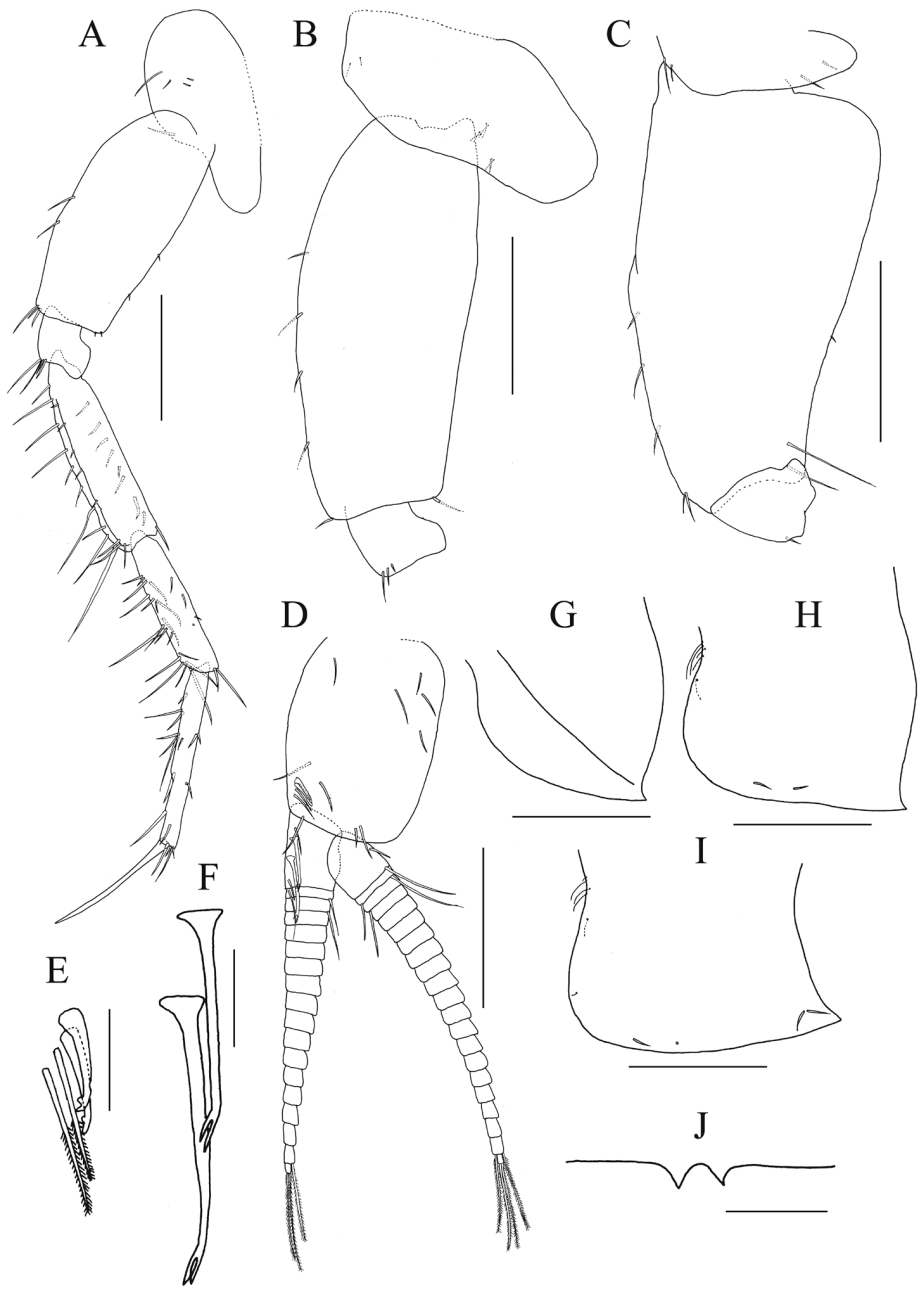
*Nicippe recticaudata* sp. n., holotype, male, 8.4 mm, NSMT-Cr 25456. **A** pereopod 5, medial view **B** coxa–ischium of pereopod 6, lateral view **C** coxa–ischium of pereopod 7, lateral view **D** pleopod 2, medial view, some setae on rami omitted **E** retinacula and associate setae on peduncle of pleopod 2, medial view **F** bifid plumose setae (clothes-pin setae) on inner basal margin of inner ramus of pleopod 2, medial view **G–I** epimeral plates 1–3, respectively, lateral views **J** dorsal margin of urosomite 1. Scale bars: 0.5 mm (**A–D, G–I**), 0.1 mm (**E, F, J**).

**Figure 5.**
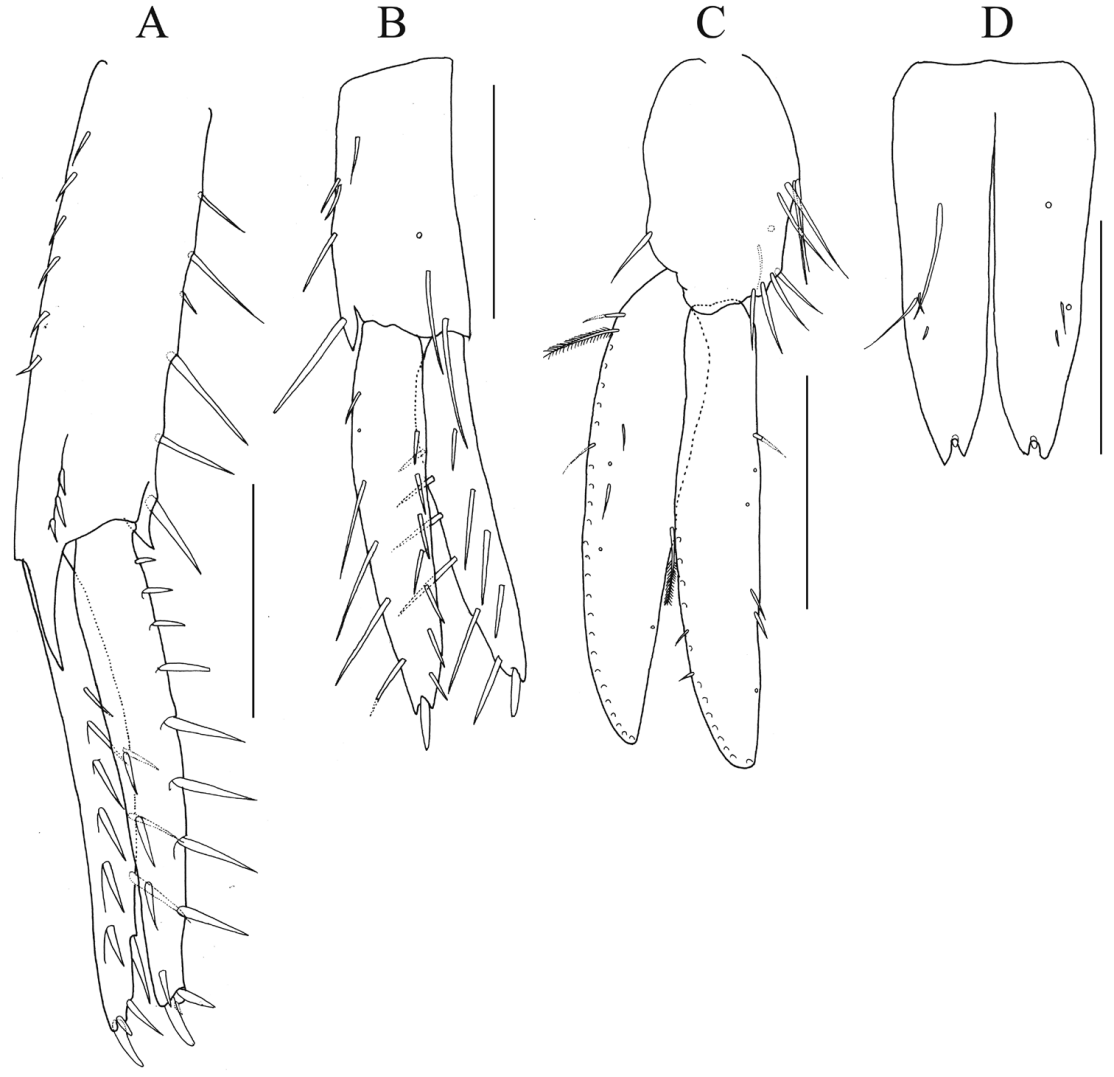
*Nicippe recticaudata* sp. n., holotype, male, 8.4 mm, NSMT-Cr 25456. **A** uropod 1, dorsal view **B** uropod 2, dorsal view **C** uropod 3, ventral view **D** telson, dorsal view. Scale bars: 0.5 mm.

**Figure 6.**
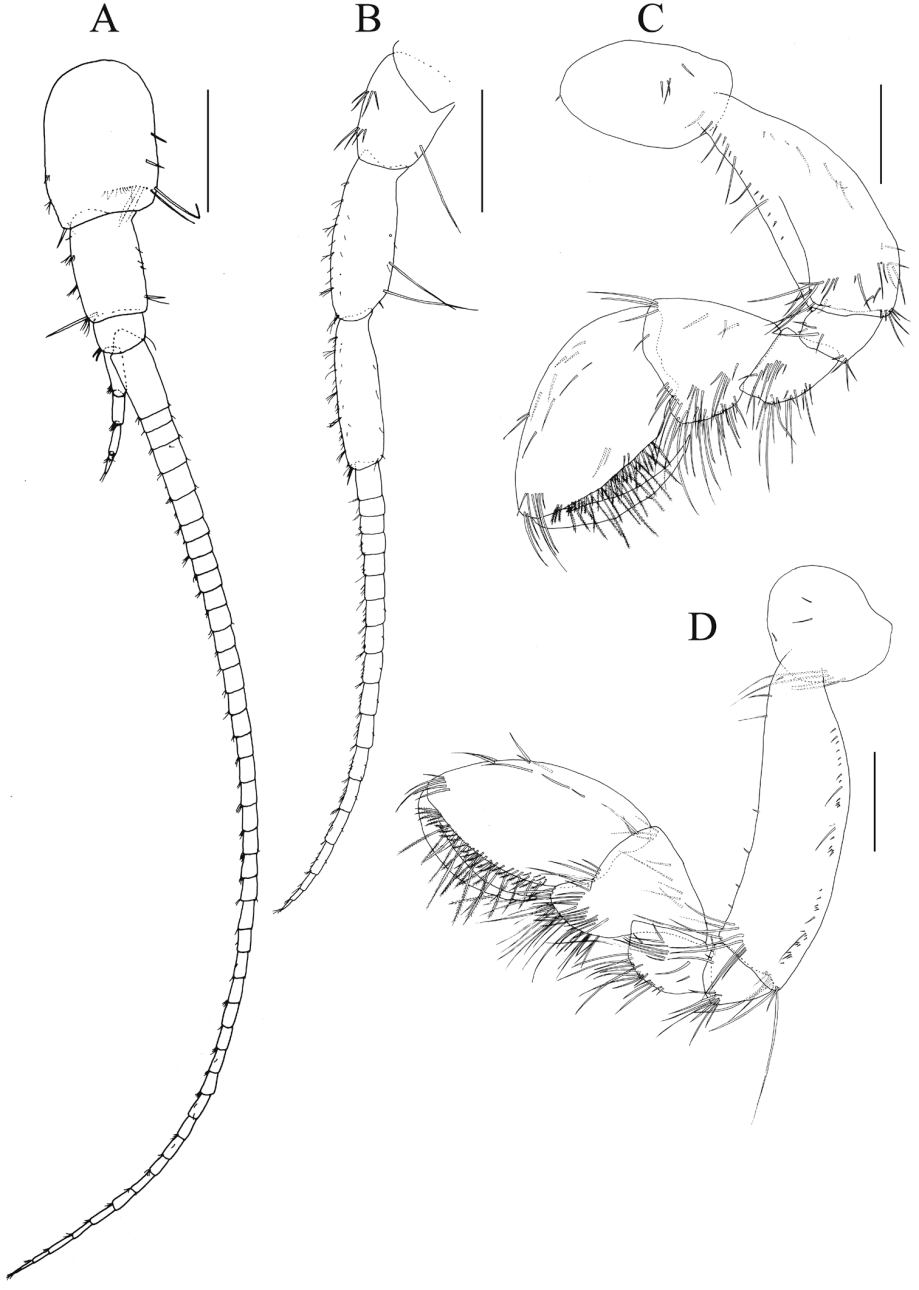
*Nicippe recticaudata* sp. n., paratype, female, 8.5 mm, NSMT-Cr 25457. **A** antenna 1, lateral view **B** antenna 2, lateral view **C** gnathopod 1, medial view **D** gnathopod 2, medial view. Scale bars: 0.5 mm.

**Figure 7.**
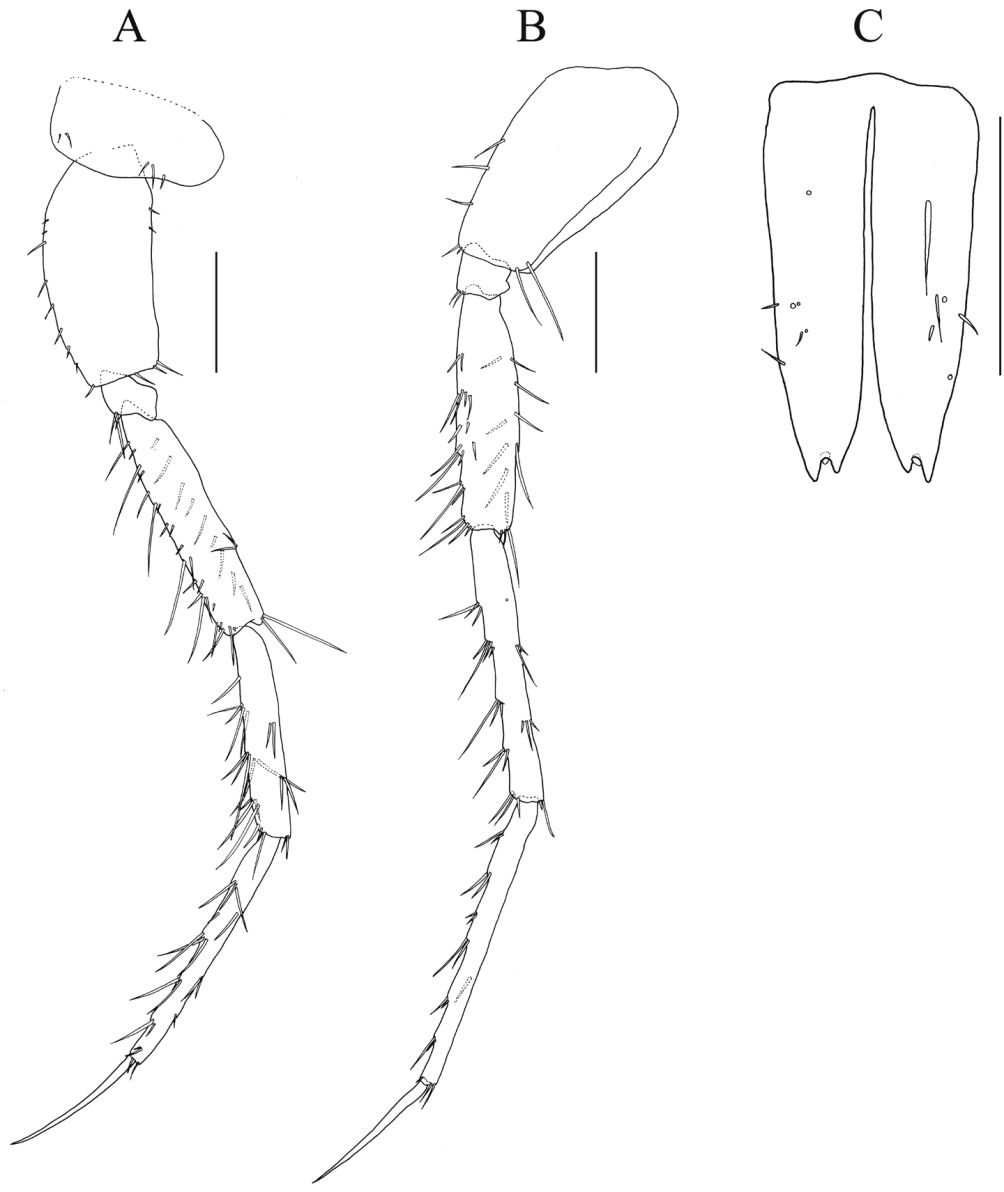
*Nicippe recticaudata* sp. n., paratype, female, 8.5 mm, NSMT-Cr 25457. **A** pereopod 6, lateral view **B** pereopod 7, lateral view, coxa lacking **C** telson, dorsal view. Scale bars: 0.5 mm.

Figures 1 , 2 , 3 , 4 , 5 , 6 , 7

Nicippe tumida : Ren 2012: 349–351, fig. 154.

###### Material examined.

Holotype: male (8.4 mm), NSMT-Cr 25456 (Fig. 1A), off Cape Toi (31°14.54'N, 131°32.20'E–31°14.94'N, 131°31.46'E; 265–367 m deep), Japan, collected by K. Tomikawa, 29 May 2006. Paratypes: female (8.5 mm), NSMT-Cr 25457 (Fig. 1B), male (8.5 mm), NSMT-Cr 25458, male (10.4 mm), KUZ Z1807, male (6.8 mm), KUZ Z1808, data same as for holotype.

###### Diagnosis.

Dorsal margin of urosomite 1 with 2 pointed teeth; posterior margin of merus of pereopod 4 with 5–6 setae; anterior margin of merus of pereopod 5 with 9–10 setae; telson with straight inner margin, tapering proximally.

###### Description of male

**(holotype, NSMT-Cr 25456).** Head (Fig. 2A) without any trace of eyes or ommatidia; rostrum pointed; lateral cephalic corners angularly produced. Pereon segments 1–4 subequal in length, slightly shorter than segment 5; segment 6–7 longest, about 1.3 times length of previous segment. Pleonites 1–3 in length ratio of 1.0 : 1.3 : 1.3. Posteroventral corners of epimeral plates 1–3 each with prominent small tooth (Fig. 4G–I); ventral submargins of epimeral plates 2 and 3 with 2 setae, respectively. Urosomites 1–3 in length ratio of 1.0 : 1.0 : 1.2; urosomite 1 with 2 pointed teeth extending beyond posterior margin of its segment (Fig. 4J); urosomites 2 and 3 dorsally smooth.

*Antenna 1* (Fig. 2B): length 0.6 × body length; peduncular articles 1–3 in length ratio of 1.0 : 0.6 : 0.3; posterodistal corner of peduncular article 1 with 2 long setae; accessory flagellum 3-articulate, article 1 long, length 4.8 × width; primary flagellum 38–articulate, length of article 1 longer than peduncular articles 2 and 3 combined, callynophore weakly developed.

*Antenna 2* (Fig. 2C): length 0.9 × antenna 1, peduncular articles 3–5 in length ratio of 1.0 : 1.1 : 1.1; flagellum 29–articulate.

*Mouthparts*. Upper lip (Fig. 2D) with shallowly concave ventral margin, lobes symmetric. Mandibles (Fig. 2E–G): slightly asymmetric, incisor margins broad, straight, anterodosal corner rounded, anteroventral corner with a strong tooth; left lacinia mobilis (Fig. 2F) broad, about 0.8 × length of incisor, multi-dentate; right incisor (Fig. 2G) with 2 teeth on proximal to anterodorsal corner; right lacinia absent; accessory setal row of left and right mandibles each with 2 robust setae, and a proximal tuft of seta; molar absent; mandibular palp 3-articulate with length ratio of 1.0 : 3.6 : 2.7, article 2 with 12 setae, article 3 with 5 posterolateral and 3 apical setae. Lower lip (Fig. 2H) with broad outer lobes, inner lobes coalesced. Maxilla 1 (Fig. 2I) with inner and outer plate and palp; inner plate small with apical seta; outer plate subrectangular with 7 spine-teeth and 1 stout plumose seta, the lateral one strongest and longest; left and right palps symmetric, palp 2-articulate, article 1 lacking marginal setae, article 2 expanded distally, with 8 robust and 8 slender setae on its apical margin. Maxilla 2 (Fig. 2J) with moderately slender inner and outer plates; inner plate bearing row of plumose setae on apical to medial margin; outer plate slightly longer than inner plate with apical plumose setae. Maxilliped (Fig. 2K) with inner and outer plates and palp; inner plate not reaching base of palp, with long plumose seta and short simple seta apically; outer plate narrowly rounded, reaching base of article 2 of palp, with setae along apical to medial margin; palp raptorial, 4-articulate, long, article 2 longest with inner marginal rows of setae, article 3 covered with 4 clusters of setae, article 4 slender with serrate inner margin.

*Gnathopod 1* (Fig. 3A): coxa ovate with seta on anterodistal corner; basis long, expanded distally, anterior margin straight, posterior margin arched; ischium short, triangular, subequal in length to merus; carpus with short rounded lobe ventrally with long setae; propodus oval, about as wide as carpus, palm straight with long setae; dactylus slender, slightly curved, inner margin smooth with tooth near the base.

*Gnathopod 2* (Fig. 3B): coxa rounded with seta on anterodistal corner, posteroproximal part with setae; compared to that of gnathopod 1, basis longer, more slender and straighter; carpal lobe stronger; dactylus similar, slightly shorter.

*Pereopod 3* (Fig. 3C): coxa quadrate, posterior margin with setae; basis long with small setae on anterior and posterior margins; merus with 8 setae on posterior margin, 7 facial setae, and a cluster of setae comprising 3 setae on posterodistal corner; carpus and propodus with plumose and simple setae on posterior margin, and laterofacial setae; dactylus slender and weakly curved, length 0.7 × propodus.

*Pereopod 4* (Fig. 3D): coxa rounded, ventral and posterior margins with setae; basis long with setae on anterior and posterior margins; merus with 5–6 setae on posterior margin, 7 laterofacial setae, and a cluster of setae comprising 3 setae on posterodistal corner; carpus and propodus with plumose and simple setae on posterior margin, and laterofacial setae; dactylus slender and weakly curved, length 0.6 × propodus.

*Pereopod 5* (Fig. 4A): coxa bilobate, anterior lobe stronger and slightly longer with seta on ventral margin; basis posterior margin slightly convex with 2 small setae, posteroventral corner subquadrate with 2 minute setae; merus, carpus, and propodus in length ratio of 1.0 : 0.8 : 0.9; merus with 9 setae on anterior margin and 5 setae on submargin, anterodistal corner with 1 long and 3 relatively short setae; dactylus slender and almost straight, longer than on pereopods 3–4, 0.76 × length of propodus.

*Pereopod 6* (Fig. 4B): coxa shallowly bilobate, anterior lobe slightly stronger; basis posterior margin slightly convex without setae, posteroventral corner subquadrate with seta.

*Pereopod 7* (Fig. 4C): coxa ventral margin shallowly concave, anterior margin and ventral submargin each with 2 setae; basis expanded more strongly than pereopods 5–6, distally tapering, posteroproximal margin convex, anterodistal margin with setae, posteroventral corner slightly produced into a small rounded lobe, armed with 2 long setae.

*Coxal gills* on gnathopod 2 and pereopods 3–5 broad, longer than those of bases; gill on pereopod 6 ovate, length 0.5 × basis; gill on pereopod 7 slender, length 0.2 × basis.

*Pleopods 1–3* (Fig. 4D) each with paired retinacula (Fig. 4E) on inner distal margin of peduncle, bifid setae (clothes-pin setae) (Fig. 4F) on inner basal margin of inner ramus; inner and outer rami of each pleopod consisting of 18 and 21 articles, respectively.

*Uropods*. Uropod 1 (Fig. 5A): peduncle slightly longer than rami, distolateral peduncular tooth very strong; outer ramus somewhat longer than inner ramus, outer and medial margins of outer ramus with 6 and 4 robust setae, respectively; outer and medial margins of inner ramus with 4 and 8 robust setae, respectively; both rami with stout setae apically. Uropod 2 (Fig. 5B): distolateral peduncular tooth short; outer ramus slightly longer than inner ramus, outer and medial margins with 6 and 7 robust setae, respectively; inner ramus with 6 outer and 4 medial robust setae, respectively; both rami with a stout seta apically. Uropod 3 (Fig. 5C): peduncle setose on outer margin, length 0.5 × outer ramus; outer ramus 1-articulate, somewhat longer than inner ramus; outer and medial margins of inner ramus each with plumose seta; medial margins of both rami with traces of setae.

*Telson* (Fig. 5D) length 2.0 × width, cleft for 88% of length in V-shape with straight inner margins of incision, each lobe with 4 setae laterally; apex of each lobe incised, lateral part of apex slightly longer than medial part, with small robust seta, lobes slightly tapering distally.

###### Description of female

**(paratype, NSMT-Cr 25457).** *Antenna 1* (Fig. 6A): length 0.6 × body length; article 1 of accessory flagellum short, length 2.4 × width; primary flagellum 43-articulate, callynophore weakly developed.

*Antenna 2* (Fig. 6B): length 0.7 × antenna 1; flagellum 18-articulate.

*Gnathopod 1* (Fig. 6C): anterior and posterior submargins of basis with many setae.

*Gnathopod 2* (Fig. 6D): anterior margin and posterior submargin of basis with many setae.

*Pereopods 6 and 7* (Fig. 7A, B): similar to those of holotype; merus–propodus slender, with setae on anterior and posterior margins, and facial setae; dactylus slender, slightly curved inward.

*Telson* (Fig. 7C): length 1.9 × width, cleft for 90% of length, incision wider than that of male, each lobe with 6–7 setae laterally.

###### Variation.

Callynophore on antenna 1 well developed (3 males, NMST-Cr 25458, KUZ Z1807, Z1808); peduncular article 5 of antenna 2 longer than 1.5 × article 4 (3 males, NMST-Cr 25458, KUZ Z1807, Z1808); posterior margin of merus of pereopod 4 with 5–6 setae; anterior margin of merus of pereopod 5 with 9–11 setae.

###### Coloration.

Color in life unknown; faded in preservative (Fig. 1).

###### Etymology.

The specific name is a compound adjective derived from the Latin words *rectus*, and *caudatus* referring to the fact that the inner margin of the telson of this species is straight, a diagnostic character of the species.

###### Sequences and COI genetic distances.

In total nine nucleotide sequences were determined: holotype (NSMT-Cr 25456), four sequences, 28S (LC214961; 1336 bp), H3 (LC214963; 328 bp), COI (LC214958; 658 bp), and 16S (LC214956; 407 bp); paratype (NSMT-Cr 25457), four sequences, 28S (LC214962; 1336 bp), H3 (LC214964; 328 bp), COI (LC214959; 658 bp), and 16S (LC214957; 407 bp); and paratype (KUZ Z1807), one sequence, COI (LC214960; 658 bp).

The obtained three COI sequences (LC214958–LC214960) were completely identical to each other. Based on the 651 bp aligned sequences, the COI uncorrected *p*-distance between *N. recticaudata* and the eastern North Pacific “*N. tumida*” was 17.1%.

###### Remarks.

Although the present specimens showed two characteristics in the degree of callynophore of male antenna 1 and the length of peduncular article 5 of male antenna 2, the obtained genetic data revealed that these morphological variants (specimens with strongly developed callynophore and long peduncular article 5 of antenna 2, e.g., KUZ Z1807 vs specimens with weakly developed callynophore and short peduncular article 5 of antenna 2, e.g., NSMT-Cr 25456) shared completely identical COI sequences, and thus they were identified as the same species. The character states of the well developed callynophore of antenna 1 and the elongate peduncular article 5 of antenna 2 were observed only in males of *N. recticaudata*. Males with these characteristics might be regarded as fully mature individuals.

According to the conventional classification of *Nicippe* species, this species would be identified as *N. tumida* based on the possession of the two-dentate posterior margin of urosomite 1. However, *Nicippe recticaudata* clearly differs from the “true” *N. tumida* defined by Bruzelius (1859) and Sars (1895) in the following three characteristics (character states of *N. tumida* in parentheses): posterior margin of merus of pereopods 4 with 5–6 setae (more than 10); anterior margin of merus of pereopod 5 with 9–10 setae (more than 20); and telson with straight inner margin, tapering proximally (sinuous inner margin, weakly expanding proximally). Moreover, the calculated COI genetic distance (17.1%) between *N. recticaudata* specimens and the *N. tumida* sample from California (Northeastern Pacific) revealed that the new species is genetically diverged from the Californian population of *N. tumida*; 3.5–4% COI distances have been proposed as the threshold for amphipod species discrimination (Witt et al. 2006; Rock et al. 2007; Hou et al. 2009). The *N. tumida* individuals inhabiting Californian waters were once reported to possess the morphological characteristics resembling those of *N. recticaudata* (Barnard 1959). Contrary to their morphological similarities, therefore, the present COI data highlighted that the population from Northwestern Pacific (*N. recticaudata*), and that inhabiting Northeastern Pacific (“*N. tumida*” in Californian waters) are different species. In summary, the present *tumida*-like Japanese population of *Nicippe* is considered as distinctive, and thus was described as a new species of the genus.

A *Nicippe* specimen identified as *N. tumida* was recorded from the East China Sea (Ren 2012). However, its description as well as figures clearly shows the diagnostic characteristics of *N. recticaudata*, and thus the Chinese sample in Ren (2012) definitely belongs to the present new species. As well, *N. tumida* has been recorded from around the Japanese Archipelago, e.g., from Sea of Japan and Sea of Okhotsk (Gurjanova 1951; Bulycheva 1957). Because these previous records lacked the detailed descriptions related to the diagnostic characters of *N. recticaudata*, their identities remain unclear. A world-wide systematic revision is essential to elucidate the cryptic species diversity in *N. tumida*.

### Key to species of *Nicippe* modified from Lörz and Schnabel (2015)

**Table:** 

1	Dorsal margin of urosomite 1 smooth	***N. buchi***
–	Dorsal margin of urosomite 1 dentate	**2**
2	Dorsal margin of urosomite 1 with 2 teeth	**3**
–	Dorsal margin of urosomite 1 with 1 tooth	**4**
3	Posterior margin of merus of pereopod 4 with many (more than 10) setae; anterior margin of merus of pereopod 5 with numerous (more than 20) setae; telson with sinuous inner margin, weakly expanding proximally	***N. tumida***
–	Posterior margin of merus of pereopod 4 with 5–6 setae; anterior margin of merus of pereopod 5 with 9–10 setae; telson with straight inner margin, tapering proximally	***N. recticaudata***
4	Pereonites 1 and 7 the widest, pereonites 2–6 shorter, subequal; uropod 3 peduncle with unarmed distodorsal lobe	***N. rogeri***
–	Pereonites 1–4 subequal, shorter than 5–7 with segment 5 the longest; uropod 3 peduncle with 3 pointed distodorsal process	***N. unidentata***

## Supplementary Material

XML Treatment for Nicippe recticaudata
